# Screening, awareness and challenges for colorectal cancer treatment in Saudi Arabia: an update

**DOI:** 10.6026/973206300200397

**Published:** 2024-04-30

**Authors:** Samer Mohammed Hassan Alqarni, Mohammed Saad Alamri, Peter Natesan Pushparaj, Irfan Rather, Zafar Iqbal, Muhammad Asif, Mahmood Rasool

**Affiliations:** 1 Department of Biological Science, Faculty of Sciences, King Abdulaziz University, Jeddah, Saudi Arabia; 2 Center of Excellence in Genomic Medicine Research, Faculty of Applied Medical Sciences, King Abdulaziz University, Jeddah, Saudi Arabia; 3 King Saud Bin Abdul Aziz University, King Abdullah International Medical Research Center, Al Ahsa, Saudi Arabia; 4 Department of Biotechnology, & ORIC, Balochistan University of Information Technology, Engineering & Management Sciences, Quetta, Pakistan

**Keywords:** Colorectal Cancer, Screening, Fecal Immunochemical Test, Treatment, Barriers, Incidents

## Abstract

Colorectal cancer (CRC) is the second most common cancer in the world. In Saudi Arabia, CRC is the most common cancer in males and the third most common in females, and its incidence rate is rising as the country continues to develop. However, the country does not have a national CRC screening program for CRC. This review aims to review recent studies that have attempted to address and rectify this issue and discern the most notable and prevalent barriers. Despite these efforts, guidelines are still lacking. Two prospective studies have been conducted in recent years, one of which was a national pilot screening program conducted by the Ministry of Health (MOH). While both had a similar number of participants, the colonoscopy rate for patients with a positive fecal immunochemical test (FIT) in the MOH program was only 20% compared to 75.8% in the Al-Kharj program. Awareness of the Saudi population regarding CRC and its screening appears to be insufficient. The most common barriers to patients' willingness to undergo screening were embarrassment, fear, and pain. Barriers to physicians are mostly related to factors outside their hands, such as lack of equipment and time. We conclude that efforts should be made to establish a national screening program and improve awareness of the population and physicians.

## Background:

**Figure 1:**
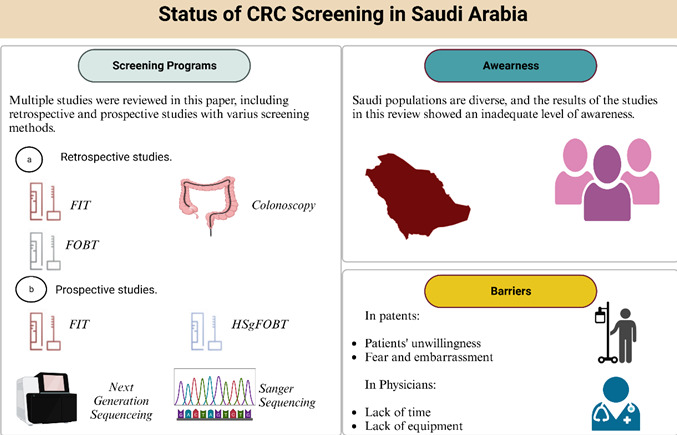
An overview of Colorectal Cancer (CRC) screening in the Kingdom of Saudi Arabia (KSA).

In 2020, colorectal cancer (CRC) was ranked third in incidence and the second most common cause of cancer-related deaths. 1.9 million New cases of CRC and an estimated 935, 000 deaths [1]. The incidence rate is significantly higher in developed countries than in developing countries; however, the mortality rates are higher in developing countries. Among the Gulf Cooperation Council (GCC) countries, Saudi Arabia has the highest age-standardized rate (ASR) among its male population and one of the lowest (ASR) among the female population [2]. In Saudi Arabia, CRC ranks first in males and third in females [3]. An increase in the CRC incidence rate for males and females in a study published in 2017 pointed to a continuous increase since 2002, along with a variation in the rates between males and females [4]. The purpose of this review was to reflect on the current state of CRC screening in Saudi Arabia, using recently published papers concerning CRC, the lack of countrywide policy, participants' awareness, and barriers (Figure 1).

## Screening guidelines in Saudi Arabia:

**Figure 2:**
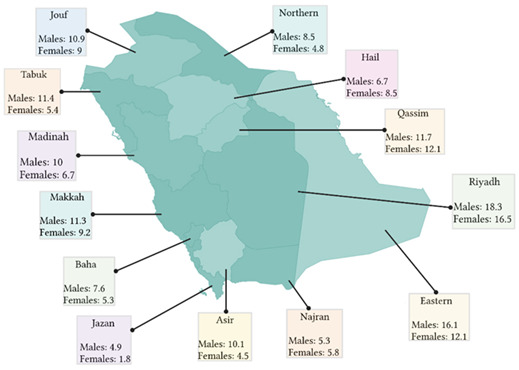
Map of Saudi Arabia showing the ASR of males and females per 100,000 people around the 13 provinces.

Currently, there is no national screening program in Saudi Arabia for CRC, despite it being the most frequent type of cancer among Saudi men and the second most frequent in women. A panel of experts was assembled by the Saudi Center for Evidence-Based Healthcare in 2015 to develop the guidelines for CRC screening in Saudi Arabia. The panel suggested initiating a CRC screening program that would target asymptomatic patients at an average risk of the disease [5]. The government of Saudi Arabia launched a national transformation program in 2016, one of the other programs aimed at fulfilling the 2030 vision. The goal of the transformation program is to reduce the prevalence of risk factors for non-communicable diseases and increase readiness for health emergencies [6]. One such effort was the establishment of the National Cancer Center (NCC) in 2017 (https://shc.gov.sa/EN/NCC/Pages/default.aspx). The goal of the NCC is to control and monitor cancer, as well as heed research and development, and to facilitate communication between the health sectors and organizations involved with cancer patients. In 2023, the NCC released a cancer incidence report for Saudi Arabia in 2020. In 2020 there were 1729 cases of CRC, accounting for 12.3% of all newly diagnosed cases. The ASR for men and women were 12.4 and 9.6/100,000, respectively. The regions with the highest ASR were Riyadh, the Eastern region, and Qassim, as shown in (Figure 2) (National Cancer Center, 2023) [7].

## Recent Screening Projects in Saudi Arabia:

However, in recent years, multiple attempts have been made to screen for CRC (Table 1). One such attempt was a pilot program that took place in 2017 throughout the country. This study was conducted using fecal immunochemical testing (FIT). 6.6 Of the 47,158 tests, 6.6% were positive and underwent colonoscopy [8] from 2017 to 2022, The Al-Kharj CRC screening program was conducted in Riyadh Province. The program used the high-sensitivity guaiac-based fecal occult blood test (HSgFOBT) as the first line of investigation, targeted at patients aged 45-75 years of age who needed colonoscopy. Almost one-third of the participating patients diagnosed with CRC had early onset cancer [9]. Most of the available studies were retrospective studies of existing screening test results. The 2022 paper published by Almoneef *et al.* (2022) aimed to test FOBT as a tool for CRC screening [10]. The study used the medical records of 2,179 patients who visited the Family Medicine Clinic at King Faisal Specialist Hospital and Research Centre in Riyadh and underwent FOBT. The study concluded that FOBT is an effective screening tool.

Other screening studies focused on the unscreened population, and another focused on surveillance colonoscopy. Alsiary *et al.* (2023) conducted a retrospective study on early onset CRC survival in unscreened populations. Early onset CRC was identified in 23.26% of the population aged 18-50 years. Late-onset in the population older than 50 years appears to have a lower rate of survival and a greater likelihood of dying compared to the significantly higher survival rate of early onset [13]. In this retrospective study conducted in Riyadh, the efficacy of surveillance colonoscopies was evaluated. Three surveillance rounds were conducted. During the first round, synchronous adenocarcinoma was detected in 0.6% of patients and metachronous adenocarcinoma in 2.6% of patients. In the second and third rounds, adenocarcinoma was identified in five out of 75 patients and in one out of 10 patients, respectively [14]. To determine the effect of sex on cancer incidence in the unscreened population, a study was performed using data from the Ministry of the National Guard Cancer Registry. Of the 1016 CRC patients in total, 37.59% were females, and 30.26% were males who had been diagnosed with metastatic CRC. Metastatic tumors appear to be 20% more likely to develop in females than in males [15]. A different screening approach was used to determine the prevalence of pathogens in patients with familial cancer, using next-generation sequencing (NGS). 47.2 Of the participants with cancer, 47.2% had CRC. This study identified 13 common variants, two of which were thought to be potential pathogenic variants. The APC c.3920T>A variant is associated with Lynch syndrome and the TP53 c.868C>T variant is associated with colon polyposis [16]. A previous study also employed NGS, Sanger sequencing, and Multiplex Ligation-dependent Probe Amplification to identify mismatch repair gene variants in individuals with Lynch syndrome. Eight high-risk cases, including four in MLH1 and four in MSH2, had variants with pathogenic or suspected pathogenic significance [17].

## Awareness of CRC screening among the Saudi Arabian population:

Many studies have been published over the years regarding the awareness and knowledge of the Saudi population regarding CRC. In a national survey covering all 13 jurisdictions of Saudi Arabia, 5720 individuals participated, 15.24% of whom had already undergone CRC screening. Males and females scored equally on knowledge, with a mean score of 11.05. The average knowledge score is the same across all Saudi Arabian jurisdictions [18]. In a survey conducted in Jeddah, a sample of 1105. Of the participants, 32.2% believed that the best age for the CRC test was 41-50 years and 25.8% responded that they did not know. Of the participants, 40.8% expressed no interest in attending CRC awareness seminars. Only 368 33.3% of the respondents were aware of any tests or examinations used to identify CRC [19]. In a survey of 909 participants in Bisha, 64.2% were over 50 years old. Most participants lacked knowledge of CRC screening, risk factors, symptoms, and diagnoses. Most patients seek medical care only if they exhibit symptoms associated with cancer [20]. Another study was conducted using an online survey of residents of Hail City to specifically study their awareness of CRC. Of the 924 participants, married individuals had lower awareness and those with a family history of CRC had higher awareness [21]. In Riyadh, 1912 residents responded to a survey on CRC. Only 51.7% of participating residents recognized the colon as the large intestine, whereas 57% knew that the rectum was located at the end of the large intestine. Although most respondents believed that colonoscopy to detect CRC early was related to high survival rates, and 72.8% stated that colonoscopy was the preferred method of screening, 65.7% preferred to avoid CRC screening [22]. This cross-sectional, observational study was conducted in Makkah, Japan. In total, 832 participants completed the questionnaire. 73.2 Of the participants, 73.2% were unaware of CRC screening. And while 1/3 of the participants were at risk of developing CRC, only 16.9% knew about screening [23]. In 2020, 1296 of the Aseer regions completed a survey on CRC. Approximately one-fifth of participants were deemed to have a good level of awareness. 95.4 Of the participants, 95.4% were willing to undergo CRC screening if they had risk factors [24]. A study on the awareness of residents of Madinah was published in 2020. Of the 385 participants, only 119 had heard of undergoing CRC screening. In general, residents had poor knowledge of CRC overall, with only 19.2% showing good knowledge and 0.9% showing exceptional knowledge of CRC [25].

## Challenges and Barriers:

## Patients' barriers:

In a national survey, 43% of the participants thought that undergoing colonoscopy would be embarrassing, and 38% thought it would be painful [18]. Multiple studies have reported fear and embarrassment as barriers among populations [19, 26, 27]. In a cross-sectional study that focused on the preferred screening methods among the population, 50% attributed their choice to how the test was performed, choosing fecal immunochemical test (FIT) as the most preferred screening method for 41.7% of the population [16]. Other barriers include a lack of knowledge about screening, as stated in a previous study [28]. However, another study found no relationship between a lack of knowledge and willingness to undergo screening [18]. Individuals living in rural areas face more barriers than those living in urban areas, mainly transportation and the unavailability of screening methods [26]. However, this study found that the lack of physician recommendations was the most common barrier among the participants. Another prominent barrier in screening is the absence of symptoms [29, 30]. The cost of screening is also a frequent reason for individuals refusing to undergo screening [3, 24].

## Physicians' barriers:

In the Al-Qassim region, physicians cited poor patient compliance, lack of equipment, lack of time, and lack of training as barriers to recommending CRC screening to patients [31]. Another study showed that physicians deal with other barriers to screening. The reported barriers include patients not following through tests and a lack of policy and reminder systems [29]. A Cross-sectional study in the eastern region of Saudi Arabia on the attitudes of physicians and nurses toward screening showed that most participants had never performed colonoscopy despite believing in its importance. Furthermore, 29% of the participants did not recommend regular screening to their patients [32].

## Discussion:

The absence of a national screening program is still a persistent issue, and there seems to be an effort to rectify it since the publication of the proposed guidelines by Alsanea *et al.* (2015) and multiple screening programs, including those by the MOH, despite its subpar results [33]. However, the national program from the MOH showed results similar to those of other screening programs in Kuwait and Qatar [34, 35]. Similarly, the retrospective studies were consistent with those of other studies conducted in the UAE, Bahrain, and Oman [36, 37, 38]. Among Gulf countries, only Qatar and the UAE have national screening programs [39], and the next-generation sequencing method of screening is still underused in Saudi Arabia, despite multiple studies on its cost and time effectiveness [40, 41]. The awareness of the population in Saudi Arabia regarding CRC and screening is inadequate, which is similar to China's high-risk population awareness; only the Chinese population was much more positive towards screening [42]. A study of rural and urban population awareness and attitudes toward CRC screening in Nebraska showed that people living in rural areas face more barriers [43]. While screening itself is not costly to the country, medication, especially personalized medicine, is [44]. The many molecular pathways involved in CRC and their heterogeneous nature pose a significant barrier to treatment. In recent years, personalized medicine has become more widely utilized than non-specific therapies such as cytotoxic agents. To best utilize personalized medicine, we must focus on screening for CRC, mutations, and hotspots [45]. In Sweden, a study was conducted on differences in treatment between screened and non-screened patients with CRC. It was found that 41% of the patients in the screening group were in stage I and underwent a more thorough multidisciplinary team evaluation than the non-screened patients. The study concluded that participation in screening reduced the need for emergency surgery [46]. The multiple studies reported in this paper showed that the Saudi population prefers the FIT screening method for colonoscopy because of fear, embarrassment, and disgust. Reynolds. (2018) found that discussing screening with a physician has a more positive impact on accepting screening than having great knowledge about CRC [47]. In the US, embarrassment is more prevalent in rural areas because of the possibility that the patient may personally know the one conducting the test [48]. This issue should be considered when focusing on patients living in rural Saudi Arabia. However, the greatest issue faced by patients in rural Saudi Arabia is a lack of healthcare [49]. The impact of physicians' advice and embarrassment was also the most common barrier among South Asians [50]. While physicians have good knowledge about CRC and screening, they lack the practice and willingness to recommend regular screening for their patients. In Switzerland, seminars for training physicians showed positive results, with physicians prescribing FIT and colonoscopy to their patients equally and frequently [51]. A physician's role goes beyond simply giving advice. In the American context, it was suggested that physicians should choose the best screening test after extensively discussing the benefits, downsides, costs, and availability with their patients [52]. Trusting physicians were reported to have a positive influence on low-income patients' attitudes toward screening [53].

## Conclusions and Future Directions:

Screening in Saudi Arabia requires more work to achieve the desired outcomes. The lack of a national screening program is the most important issue to address in the national transformation program. Population awareness is a key factor in the success of screening programs; therefore, educating the population about CRC and screening is vital. Based on existing studies, physicians have good knowledge of screening; however, they require first-hand experience with colonoscopy. In America, there is an aim for the CRC screening rate to reach 80%, which was believed to lower the incidence rate by 17% in 2018 [54]. Further efforts are suggested to use videos and websites to educate people on CRC and screening, mail to deliver FIT to patients and reminders for healthcare providers on upcoming tests [55].

## Figures and Tables

**Table 1:** This table shows the various screening programs and retrospective studies that have used colonoscopy and stool-based tests in the last few years.

**Author**	**Year**	**Study design**	**Setting**	**Screening Method**	**Results**
Zacharakis *et al.* [9]	2023	Prospective study	A screening program was implemented from 2017 to 2022 in three hospitals in Al-Kharj.	HSgFOBT	The study included 35,640 participants, 51% of whom were males. The test had 6.3% positive results, and 75.8% of them underwent colonoscopy. PDR* and ADR** were 33.3% and 25.4% respectively. Colon cancer was present in 4.8%.
Almoneef *et al.* [10]	2022	Retrospective study	Records of patients above 50 who visited King Faisal Specialist Hospital and Research Centre between 2002 and 2017.	Immunological FOBT	2,179 patients were included, and 19.7% had positive results. After the positive result, the Colonoscopy rate was 52%, with PDR being 47.9% and ADR 34%. 3.5% of the colonoscopy tests showed Colon cancer.
S. Alharbi *et al.* [11]	2022	Retrospective study	A cross-sectional study of patient records from 2010 to 2020 in Alnoor Specialty Hospital, Makkah.	colonoscopy	2,158 cases were included in this study, 55.4% of whom were males. Colon cancer was 8%, with PDR being 14%. Tumor and bleeding, polyp, and hemorrhage were found to be statistically associated.
MoH Pilot Project	Not Published	Prospective study	National screening program implemented in 417 centers around the country.	FIT	Including 47,158 the FIT resulted in 6.6% positive tests. Of those with positive results, only 20.4% underwent colonoscopy. The PDR was 32.4% and the Colon cancer was 7.2%.
M. Almadi *et al.* [12]	2019	Retrospective study	The study uses the medical reports stored in the database of three hospitals in Riyadh, between 2016 and 2017.	Colonoscopy	The study included the records of 1,180 patients. The PDR and ADR were 24.8% and 16.8% respectively. Polyps were distributed as follows, sigmoid colon 28.3%, rectum 22.0%, ascending colon 11.2%, and cecum 10.3%. Colon cancer was present in 1.6%.
*Polyp deduction rate
**Adenoma deduction rate
